# In plain sight: implicit priming of patterns and faces using change symmetry

**DOI:** 10.1007/s00426-020-01434-w

**Published:** 2020-10-23

**Authors:** Aleksandar Aksentijevic, Finbar Duffy, Anja Mihailovic, Dragutin T. Mihailovic

**Affiliations:** 1 Department of Psychology, University of Roehampton, Whitelands College, Holybourne Avenue, London, SW154JD UK; 2 Department of Psychological Sciences, Birkbeck, University of London, London, UK; 3 Independent Researcher, Novi Sad, Serbia; 4 Faculty of Natural Sciences, Department of Physics, University of Novi Sad, Novi Sad, Serbia

## Abstract

Aksentijevic–Gibson complexity is an original complexity measure based on the amount of change in a string or 2D array that has been successfully implemented on data from psychology to physics. The key ingredient to computing the measure is a change symmetry (CS)—a novel form of structure (also known as generalised palindrome) which represents a central or mirror symmetry based on the redundant arrangement not of symbols but of changes. This results in patterns that although globally symmetrical do not appear as such when inspected locally. We used this property to (a) affect the registration of a target, (b) prime the symmetry judgment of 2D arrays and (c) faces using 1D patterns possessing change symmetry. In Experiment 2, we applied the lock and key principle to complete the prime without showing its structure at once. In Experiments 3 and 4, we presented subjects with fast sequences of CSs such that the configuration of an individual pattern was masked by the subsequent pattern leaving only the structural “essence” of the prime symmetry. The results strongly support the contention that higher-level hidden structure of change symmetry successfully primes the symmetry perception of 2D arrays as well as facial attractiveness.

## Introduction

The study of pattern structure has long been an important facet of psychological research. Issues of perceptual organization, structuring of information and mechanisms of structure processing have occupied researchers since the dawn of psychological science, especially in the context of experimental aesthetics (Fechner, 1876; Wundt, 1898). This line of research received a substantial impetus from the perspective of Gestalt psychology (e.g. Koffka, 1935), which focused on the relational aspects of perception and cognition. Somewhat later, the introduction of the Information theory (Shannon, 1948) revived interest in the relationship between pattern and information especially through the work of Attneave (1954, 1959) and Garner (e.g. 1974). Introduction of statistical methods into the study of pattern structure offered a promise of precise quantification of structural relationships within a pattern. Despite substantial interest in psychological complexity starting in the 1950s, progress has been slow (see Luce, 2003; Simon, 1972 for reviews) principally because traditional methods fail to capture the structural/relational properties of the pattern.

Investigations of pattern complexity in the 1970s and 1980s have provided evidence that processing of pattern structure has a hierarchical character—not unlike other processing domains (Alexander & Carey, 1968). Researchers, such as Chipman (1977), put forward a hypothesis according to which pattern processing is governed by two processes. One is primarily concerned with the quantitative aspects of a pattern—the number of elements or element clusters—whereas the other is responsible for processing pattern structure. Later work by Ichikawa (1985) demonstrated that the first process is fast and that the second requires more time. Quantitative processes focus on pattern elements, such as number of dots, runs or clusters. In contrast, structural processes are concerned with detecting periodicities, symmetries and other structural relationships. Consequently, Ichikawa proposed a two-stage model of structure processing. He named the first stage “primary” and the second “cognitive” linking them to processing time. With short stimulus presentation times, quantitative factors dominate the judgment and the structural aspects are discovered only at a given sufficient time.

One of the most important factors in determining complexity is symmetry (Weyl, 1952), a highly salient form of redundancy which consists in a repetition of a pattern which appears to disobey the rules of perceptual integration. Symmetrical repetitions can be singular or multiple, translational, mirror or rotational (Treder, 2010; Wagemans, 1997). The prototypical form of symmetry is mirror or central symmetry which can be decomposed into repetition plus rotation of the duplicated part by 180° horizontally. When the two halves are joined, we recognise a form of redundancy which is not only common in nature (plant, animal and human body shape) but is also ubiquitous in production, design and art. Mirror or central symmetry is highly salient and desirable and this has led to a number of theories linking it to the fundamentals of human existence, such as energy conservation and genetic inheritance. The special status of symmetry in perception has been confirmed both experimentally (Huang, Pashler, & Junge, 2004) and neurophysiologically (Sasaki, Vanduffel, Knutsen, Tyler, & Tootell, 2005), in that its processing does not require attention and is confined to a specific brain region. The question we wish to address in this paper is: is a higher, more abstract form of symmetry possible which is not noticeable even under attention? If so, would this kind of symmetry still impact the perception of related and unrelated qualities?

Recently, Aksentijevic et al. reported a complexity measure which connects the subjective perspective of the human observer and the third-person perspective of mathematics and science by acknowledging the importance of the processing cost (Aksentijevic–Gibson complexity; AG) (Aksentijevic, Mihailovic, & Mihailovic, 2020; Aksentijevic, Mihailovic, Kapor, Crvenkovic, Nikolic-Djoric, & Mihailovic, 2020; Aksentijevic & Gibson, 2012a, b). In contrast to current theories, Aksentijevic–Gibson complexity offers a simple, unifying definition of complexity, namely, change. Study of changes in sensation forms the foundation of psychology. Any account of sensory processing highlights the importance of change in parameters for stimulus encoding. Similarly, there is overwhelming evidence that the brain is primarily attuned to processing change. After all, the function of neurons is the registration of change in a binary manner. Change connects psychological, physical and computational meanings of information/entropy. Any perception, cognition or action must involve change as well as irreversible conversion of energy. Change equals increase in entropy, and this in turn equals cost. Unlike invariance, change can be easily defined and, while invariance can be easily described in terms of change, the converse is not straightforward (Cutting, 1998). Change allows direct quantification of the relationship between pattern elements. Structural information is contained in the transition from one symbol (or element) to another and not in the symbols themselves.

We tested AG using a large number of complexity-related data reported in the literature including subjective and objective complexities, subjective randomness, subjective and objective symmetry, subjective goodness, mean verbalization length, informational entropy, syntely, subsymmetries, partial symmetry, SIT information index, tapping variability, copying accuracy, memorization, sequential prediction, symmetropy and rhythm reproduction accuracy and variants of Kolmogorov complexity. We tested our measure on sequences and arrays, visual and auditory patterns, and without exception obtained significant correlations with data spanning 50 years of research. In contrast to other measures, our measure successfully models statistical properties of subjective complexity judgments. In addition, we were able to explain and quantify the changes in perceived complexity as the function of stimulus exposure time.^1^ Besides generality and simplicity, the most valuable property of our model is the fact that it quantifies complexity at all structural levels. This allows us to examine subjective complexity performance in much more detail than possible with other measures and to investigate different levels of the structure-processing hierarchy. For example, we can model the quantitative stage with weights that favour short substrings of S, and the structural aspect with weights that favour all lengths equally (Aksentijevic, Mihailovic, Kapor, et al., 2020; Aksentijevic & Gibson, 2012b, Sect. 3.1).

As already stated, the basis of AG is the change profile which is obtained by scanning a string exhaustively (i.e. with maximum overlap) using windows of increasing length and tallying changes at each level. Unlike Shannon entropy, AG complexity scans the string completely redundantly (taking into account all overlaps), that is, the scanning window moves by one step at a time irrespective of size. For a string

$$0{1}0{11}00{1}00{1}0{1},$$

we scan for changes between individual symbols, pairs, triplets… all the way to level *L* − 1. This results in a directional tally of changes for all levels of the string (from left to right):

$${96486655432}0$$

There are nine changes at the level of symbols, six at the level of pairs and so on. The complexity of the string is computed by multiplying individual entries by the weighting factor ${w}_{j}=\frac{1}{L-j}$ and summing the products.^2^ Thus, the AG complexity $(C)$ for the above string is

$$C=\sum_{j=1}^{L-1}{p}_{j}{w}_{j}=9.02,$$

where $L$ is string length (in this calculation $L=12$ and ${p}_{j}$ is the number of changes at level $j$). In this example, $j$ = 1, 2, 3… 12. Levels $j$ are reciprocals of the number of possible scans at a particular level. For example, there are 12 possible scans at $L$ = 1 and only one at $L$ = 11. AG complexity is intimately connected to symmetry (Aksentijevic, Mihailovic, Kapor, et al., 2020). In the course of our work, we noticed that the efficiency of our measure in detecting pattern regularities was related to a hitherto unacknowledged form of palindromicity we call change symmetry (CS; also generalised palindrome). In normal usage, a palindrome is a sequence of characters which reads the same in both directions. In other words, palindromic strings possess central or mirror symmetry (e.g. 123454321). More generally, a string S can be called an alphabet palindrome (AP) if its reverse is either S or the complement of S. Finally, a change symmetry generalizes the concept of palindromicity even further—in terms of change. An AP of $L > 2$ is a CS. The converse is not true for strings $L \ge 9$. Informally, CSs are patterns that register no-change at the highest structural level ($L - 1$). This means that read from left to right and vice versa, the change profiles of the longest substrings are identical despite the fact that the pattern does not appear palindromic.

Applying the formula to the above string, we obtain:

$$C=\sum_{j=1}^{L-1}{p}_{j}{w}_{j}= 9/12+6/11+4/10+8/9+6/8+6/7+5/6+5/5+4/4+3/3+2/2+0/1= 9.02.$$

As can be seen, at the very last level ($L$ = 12; rightmost fraction), only one comparison is possible. This arrangement gives AG both theoretical substance (complexity is a hierarchical phenomenon) and it also lends flexibility to the measure—although the number of changes is fixed, the weighting at different substring lengths are open to adjustment—similar to the way in which human perception focusses on various information levels, depending on the context.

**Fig. 1:**
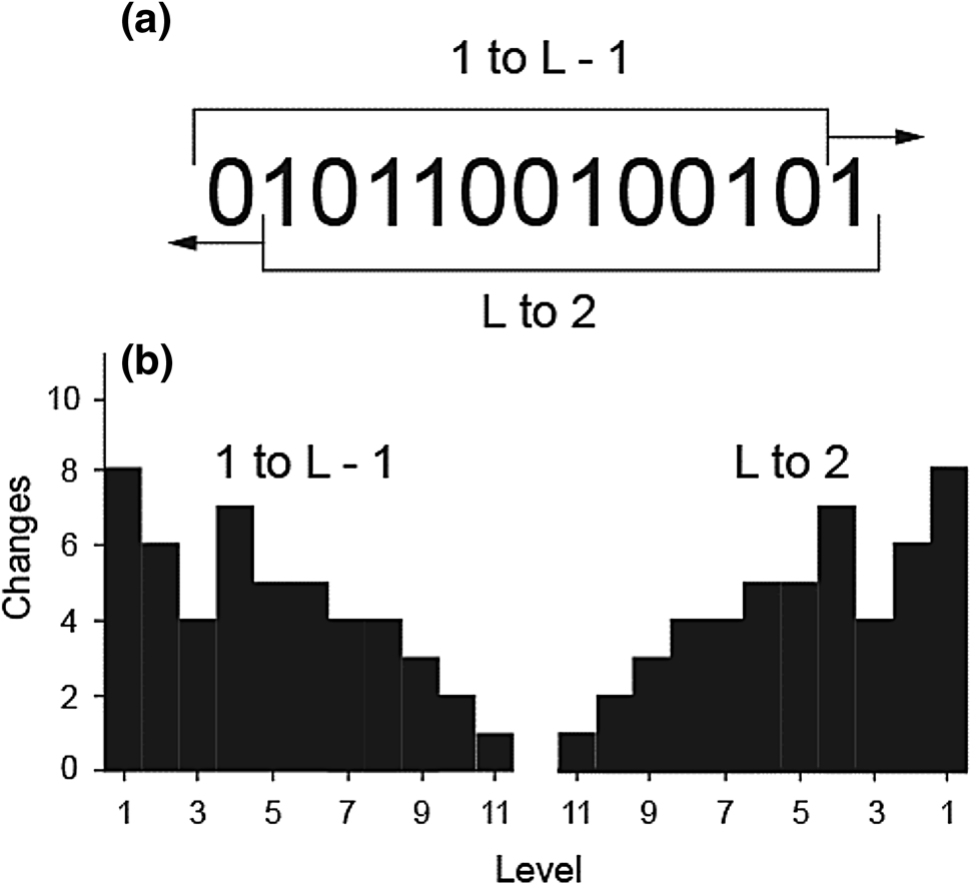
Structural schematism of change symmetry for string 0101100100101. **a** The two longest substrings are complements. **b** They possess identical (and symmetrical) change profiles and this gives rise to a kind of a global symmetry. The direction of scanning is irrelevant but is given to relate CS to mirror symmetry. The (reasonable) assumption is that the string is scanned in parallel

In the above string, the two substrings to be compared are 010110010010 (1 to $L$ − 1) and 101100100101 ($L$ to 2). The two substrings possess the same profile: 86475544321 and are, therefore, identical in terms of change. Figure 1 makes explicit the equivalence between the two substrings as well as the mirror symmetry of the two change profiles. When the whole string is observed, local asymmetry is in conflict with the global change symmetry (Fig. 1). Let us look at the above string from both ends simultaneously. 01 and 10 possess a single change. 010 and 101 contain two changes each. 0101 and 1010 contain three changes each at level 1 and no changes at level 2 all the way to $L - 1$. In this sense, CSs are fully palindromic in terms of change. Thus, the critical difference between mirror symmetry and change symmetry is that the unit of comparison is not a pattern element (or a group of elements) but the relationship between elements.

This form of hidden symmetry opens up the possibility of dissociating perceptual and neural aspects of symmetry processing.^3^ It is possible that the brain responds to hidden structure by reducing its energetic demands despite observers’ inability to distinguish CSs from other patterns. The aim of the present study was to investigate the ability of CSs to dissociate overt and covert aspects of pattern structure processing. In other words, it is hypothesized that CS will implicitly prime symmetry perception despite not being discriminated overtly from similar patterns lacking this property. The aim of the current paper is to examine three questions. First, does the structure embedded within CSs affect perception implicitly? Second, can CS prime facial attractiveness? Finally, can 1D symmetry prime the perception of 2D stimuli? While the first two questions have not been posed before, there is some evidence that symmetry can be primed if the primes and targets are of the same dimensionality (Yamauchi, Cooper, Hilton, Szerlip, Chen, & Barnhardt, 2006).

## Experiment 1: time course of overt CS registration

As a first step, we needed to confirm that CS was perceptually indistinguishable from non-CS patterns. Since this is assumed to be a function of exposure time, an appropriate time window had to be found within which this would obtain. A pilot experiment showed that with 3000-ms presentation time participants were able to distinguish CSs and non-CSs in terms of complexity.^4^ Consequently, it was decided that presentation times of 750 and 1500 ms should be used.

### Methods

#### Participants

The study received approval from the Ethics Committee of the Department of Psychology, University of Roehampton. 23 volunteers (14 female) completed the rating task (mean age 23 years, SD 6.51 years). They were recruited via email contact and social media posts on university pages. Four participants were left-handed and all had normal or corrected-to-normal vision. As in the remaining experiments, they all signed a consent form and received no remuneration for their participation. Sample size could not be controlled because the experiment was run online and (see “Procedure”).

#### Design, stimuli and apparatus

The experiment employed a 2 × 2 repeated-measures design with factors duration (750 ms, 1500 ms) and change symmetry (present, absent).

10 CSs of length 13 were selected from the 232 available patterns such that they contained more than 6 runs (single or grouped elements of the same value).^5^ The Individual CSs differ in the number of runs (uninterrupted sequence of a character/number)—from 4 (0000010011111) to 10 (0101000110101). The selected strings had between 6 and 10 runs. $L$ = 13 was chosen because preliminary trials have shown that longer strings are difficult to process (see Appendixes 1 and 2 for the exhaustive listings of CS classes with $L$ = 13 and 14, respectively). Then, the patterns were manipulated as follows: a symbol, pair or triplet of symbols (adjacent or non-adjacent) was shifted left or right so that the change symmetry (as defined by Aksentijevic and Gibson (2012a) was abolished without changing the number of runs. This produced 10 non-CS strings which while appearing similar to the CS strings lacked general palindromicity (see Table 1). It also ensured that CSs and non-CSs had the same luminance values.

**Table 1:** Ten change symmetries (*L* = 13) and their non-CS analogues used in Experiment 1 complexity values (AG) have been calculated from Aksentijevic and Gibson (2012a)

CS string	AG	Analogous non-CS string	AG	Runs
0101000110101	8.65	0100100110101	9.91	10
0100110001101	9.01	0101100001101	9.49	8
0010000111011	8.84	0010001110011	9.85	6
0001001011000	9.11	0001001001100	9.82	7
0100011001101	8.77	0100011011001	9.92	8
0101100111010	8.91	0100110111010	10.16	9
0101110000101	8.85	0101110001001	9.95	8
0001001100111	8.60	0001011000111	9.81	6
0011010010011	9.04	0011001001011	9.96	8
0001010010111	8.88	0010100100111	10.00	8

**Fig. 2:**
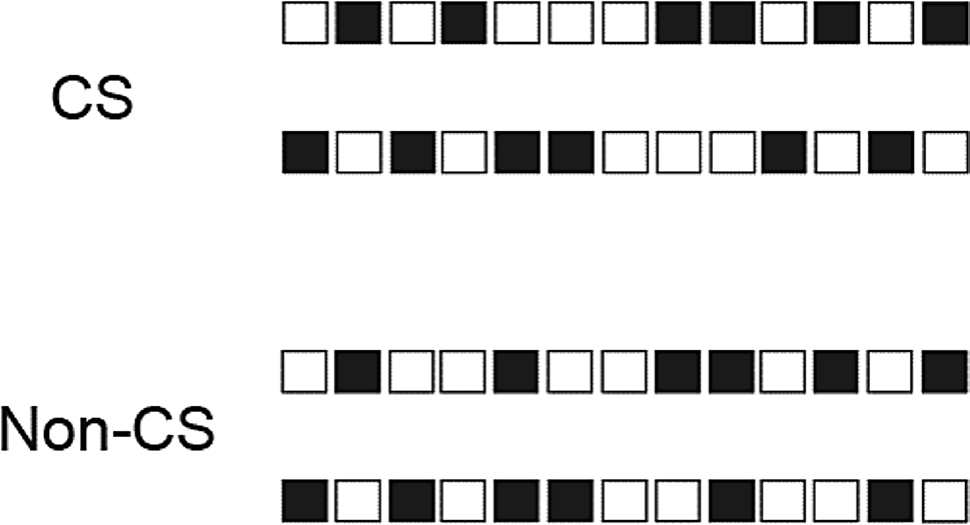
Examples (two of each) of CS and non-CS stimuli presented in Experiment 1

These patterns were then drawn in the form of horizontal bars consisting of black and white squares separated by a 2-mm gap (Fig. 2). On-screen dimensions were 98 × 7 mm. As predicted, the disrupted patterns were significantly more complex as confirmed by an independent-samples *t* test, $t$(18) = 13.59, $p$ < 0.001. These patterns were used to obtain complexity ratings. 20 stimuli (10 CSs and 10 non-CSs) were shown in two presentation time conditions giving 40 stimulus displays per participant.

#### Procedure

Viewing conditions were not controlled because the experiment was conducted online using Qualtrics software. At the start, each subject signed an online consent form, completed a short demographic questionnaire and read the instructions. Their task was to rate each stimulus for complexity on a scale from 1 (very simple) to 6 (very complex). “Complexity” was chosen because (a) it carries a distinctive meaning and (b) cannot be replaced by the concepts, such as “symmetry” (too narrow”) or “order” and “goodness” (too broad). Stimuli were fully randomized across the experimental session which was initiated by selecting a start button at the bottom of the page. At the offset of each stimulus, a rating scale appeared in the form of a multiple-choice row with six radio buttons denoting different levels of agreement. Once a response was made, a 1500-ms delay preceded the next stimulus. At the end of the session, participants were debriefed and thanked for their participation. An individual session lasted no longer than 10 min.

### Results and discussion

**Fig. 3:**
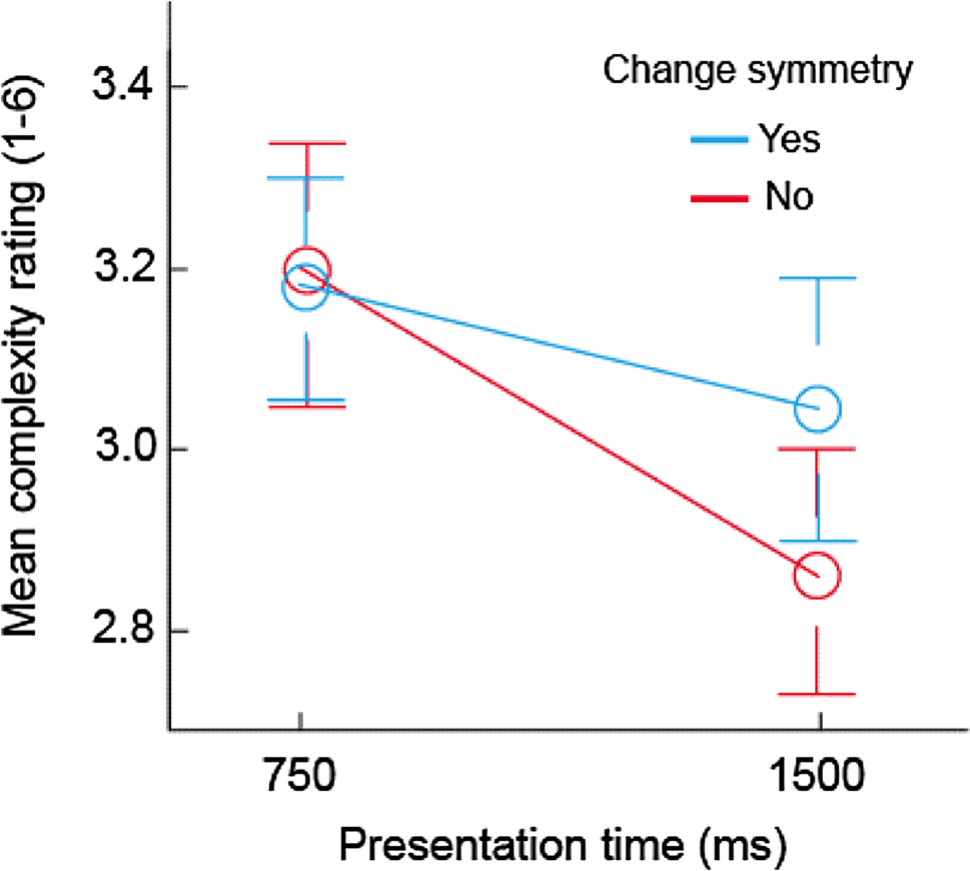
Mean complexity ratings as a function of presentation time and change symmetry in Experiment 1. Bars denote ± 1 SEM

A 2 × 2 repeated-measures ANOVA was performed on mean complexity ratings. The main effect of symmetry was nonsignificant, *F*(1, 22) = 0.79, *p* = 0.41, *η*_*p*_^2^ = 0.03, indicating that overall, the two types of pattern were not discriminated. However, a significant main effect of presentation time, *F*(1, 22) = 7.02, *p* = 0.015, *η*_*p*_^2^ = 0.24, confirmed that perceived complexity decreased with exposure. Finally, a significant change symmetry by presentation time interaction, *F*(1, 22) = 4.71, *p* = 0.041, *η*_*p*_^2^ = 0.18, reflected the fact that CSs were judged less complex at 1500 ms but not at 750 ms (Fig. 3).

Experiment 1 demonstrated that complexity of the presented patterns decreases with exposure. This finding confirms that the complexity of a pattern is partly a function of the time available for inspection. As noted by Aksentijevic (2015), the more time there is to analyse a pattern (or a process), the less complex it appears. More relevant to the aims of the present study was the finding that with short exposure, CSs could not be distinguished from non-CSs. While it would be premature to speculate on the time needed for the change symmetry to become available to consciousness, a preliminary estimate would be of the order of 1000 ms (for the given stimulus parameters). It should also be noted that a difference in ratings cannot be taken as conclusive evidence that the symmetrical nature of CSs was explicitly detected at 1500 ms—only that the difference in complexity indexed by AG (see Table 1) became salient. This is supported by an absence of correlation between AG complexity and subjective ratings, (which was expected because of relatively short exposure times which favour low-level patterning).

## Experiment 2: the “lock and key” priming of 1D symmetry

**Fig. 4:**
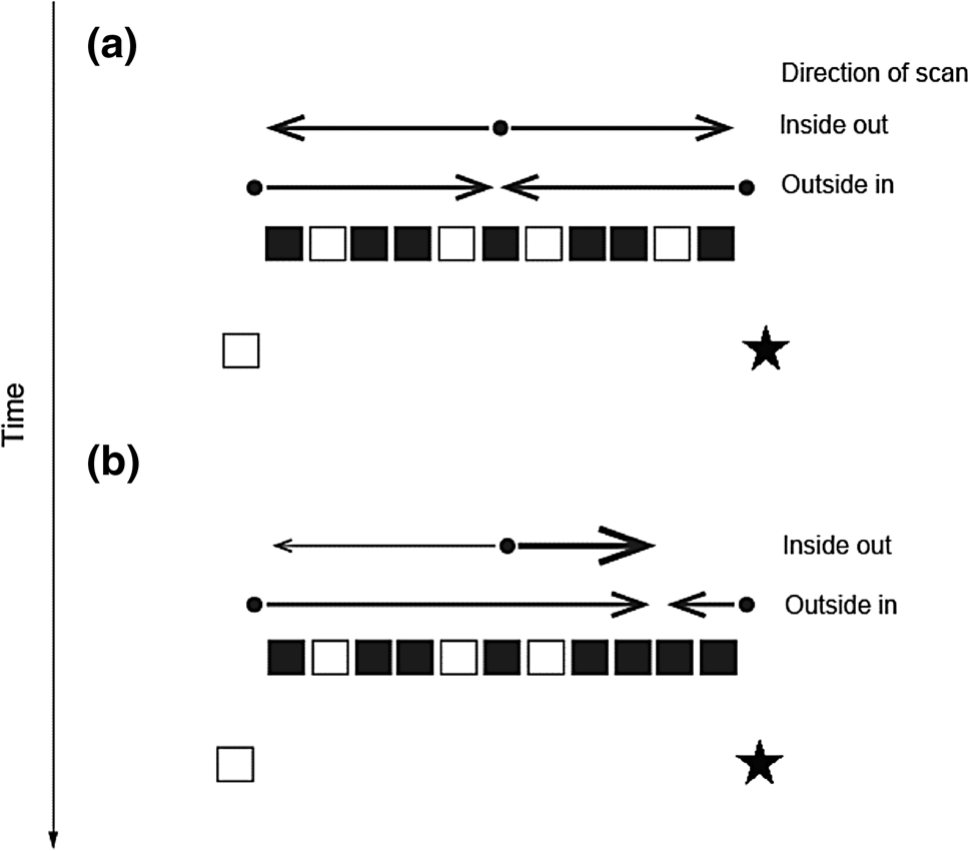
Potential effects of symmetry on attention. **a** A symmetrical pattern does not facilitate lateral biases unlike, **b** asymmetrical patterns which can abolish response equiprobability due to the presence of salient features on one side. Arrow thickness corresponds to the probability of response

Following Experiment 1, Experiments 2, 3 and 4 were planned and executed in the laboratory within a single session with the aim of testing how different CS priming techniques affected different aspects of symmetry processing. If CS is a form of symmetry, we could expect it to affect spatial perceptual judgments. To illustrate if a priming stimulus is laterally biased (asymmetrical), it might guide perception towards or away from the target which is located to the left or the right of it 50% of the time. By contrast, if the prime is perceived as symmetrical, the system will require longer time to decide which side to select. Equally, if the gaze is fixated on one side, a central symmetry might impede its shift to the other side. Figure 4 illustrates this assumption. If a priming stimulus is symmetrical and the fixation is central (a, inside out), there is no preferred direction to which gaze might be attracted. By contrast, if the prime is asymmetrical, it would draw attention to any salient feature (e.g. black cluster in b, inside out). If the gaze is fixated on the edge element of the prime, it will be attracted to the centre in case of symmetry (a, outside in) or again, to any salient features (b, outside in). This results in a bias for non-symmetrical primes (a 25% information gain) if the target is located on the same side as the salient feature. We hypothesised that CS primes would behave like visibly symmetrical stimuli and offer no lateral advantage relative to asymmetric stimuli.

Imagine a typical priming trial in which a prime is replaced by a target—a standard paradigm that has been used in psychology from its beginnings. The problem with using this kind of method to prime symmetry judgments is that the symmetry of the prime (or absence thereof) is very salient and difficult to mask. The local asymmetry of CS can be used profitably here. We have seen from Experiment 1 that a CS is recognised if presented at 1500 ms. Rather than presenting a single CS and hoping that it has lasted long enough to prime the target (and risk invalidating the experiment), we decided to use a novel priming method. Having selected 12 CSs of length 13 and having disrupted these to obtain 12 non-CS strings, we divided each string into an 11-element “lock” and two-side elements (“key”). If the former is presented first, its CS is completed only once the latter is added. Thus, both components of the pattern are necessary to perceive CS. We call this “lock and key” priming in that the two-side elements act as a key to (a) “fit into the lock and (b) “unlock” the hidden symmetry. Only when both parts of the prime have been presented can the CS be primed.

You will notice that all locks are laterally biased—they possess no central symmetry and consequently “pull” towards left or right (shaded segments in Table 2). The same is true of the completed primes—whether CS or non-CS. We assume that target registration is affected by this bias, in that the eye might be predisposed to favour one of the sides. However, given that any systematic bias was removed by counterbalancing and randomisation, no effects of accuracy were expected. In other words, both hit and false alarm rates would be equally affected. Consequently, we predicted that the presence of CS would slow down a target side decision by countering the feature-related bias and increasing the uncertainty about target location—without affecting response accuracy. Since we do not currently understand the underlying mechanism, we would equally correct in saying that non-CS patterns speeded up responses. Following the results of Experiment 1, we predicted that this effect would be observable at 1500 ms—after the lock has had sufficient time to embed itself in the memory and prime the symmetry response.

**Table 2:** Patterns and counterbalancing in Experiment 2

Pattern	CS	Runs	Black side	Star side	Target
0**00010110011**1	0	6	R	L	WL
0**00010110011**1	0	6	R	L	WL
0**00110100011**1	0	6	R	L	WL
0**01110010001**1	0	8	R	R	BR
0**01011001001**1	0	8	R	R	BR
0**01101000101**1	0	8	R	R	BR
1**11011000110**0	0	6	L	L	BL
1**11011000110**0	0	6	L	L	BL
1**11001100011**0	0	6	L	L	BL
1**10010011110**0	0	8	L	R	WR
1**10010111010**0	0	8	L	R	WR
1**10010110011**0	0	8	L	R	WR
0**00100110011**1	1	6	R	L	WL
0**00110010011**1	1	6	R	L	WL
0**01110100001**1	1	6	R	L	WL
0**01011000101**1	1	8	R	R	BR
0**01101001001**1	1	8	R	R	BR
0**11100101000**1	1	8	R	R	BR
1**11011001100**0	1	6	L	L	BL
1**11001101100**0	1	6	L	L	BL
1**10001011110**0	1	6	L	L	BL
1**10100111010**0	1	8	L	R	WR
1**10010110110**0	1	8	L	R	WR
1**00011010111**0	1	8	L	R	WR

CS = change symmetry (0 = no, 1 = yes); black side: side on which a black symbol appears, irrespective of shape; star side: side on which a star appears irrespective of colour; target: Colour and location of the star target. The central “lock” segments are highlighted in bold

### Methods

#### Participants

25 participants recruited via the departmental online subject allocation system took part in Experiments 2, 3 and 4 (14 males; mean age 23.20 years, SD 5.26 years). All participants were right-handed and all had normal or corrected-to-normal vision. All participants gave informed consent and received book vouchers for their participation. Order of experiments was counterbalanced by means of the Latin Square design. Sample size was selected based on an a priori power calculation (Table 3).

**Table 3:** Parameters of 12 change symmetries (*L* = 13) and their non-CS analogues used in Experiments 2, 3 and 4

CS string	AG	Analogous non-CS string	AG	Runs
0001001100111|	8.60	0000101100111	9.49	6
0001100100111	8.84	0001101000111	9.62	6
0011101000011	9.03	0011100100011	9.48	6
0010110001011	8.83	0010110010011	9.84	8
0011010010011	9.04	0011010001011	9.69	8
0111001010001	8.92	0111001001001	9.75	8
1110110011000	8.60	1110110001100	9.75	6
1110011011000	8.84	1110011000110	9.29	6
1100010111100	9.03	1100100111100	9.80	6
1101001110100	8.83	1100101110100	9.69	8
1100101101100	9.04	1100101100110	9.52	8
1000110101110	8.92	1001100101110	9.93	8

*AG* Aksentijevic–Gibson complexity

#### Design, stimuli and apparatus

The design was a 4-way repeated-measures design with factors “lock” duration (750, 1500 ms), star side (left, right), black side (left, right) and CS (present, absent). The stimuli were 12 preselected CS strings of length 13 which were disrupted to produce 12 analogous non-CS strings. For this experiment, 12 CS patterns possessing either 6 or 8 runs and terminating with different symbols (0 and 1) were selected. The original stimulus set was complemented so that there was an equal number of patterns starting with 0 and 1 on left or right (see Table 2). The constraint in terms of runs led to the use of complements. The second sextuplet of CS strings are complements of the first six. This was not considered critical because from feature standpoint, the complements are easily distinguishable. Change symmetry was disrupted as in Experiment 1, producing 12 non-CS strings (see Table 1). Again, the disrupted non-CS strings were significantly more complex relative to the CS strings, $t$(22) = 11.29, $p$ < 0.001.

These patterns were then drawn in the form of horizontal bars consisting of black and white squares separated by a 2-mm gap. This resulted in a fully balanced set of 24 priming stimuli (12 CS and 12 non-CS). The screen dimensions of the complete stimulus ($L$ = 13) were 98 × 7 mm which subtended 7 × 0.5° of visual angle from the viewing distance of 80 cm. In all three experiments, the stimuli were presented on an Iyama Vision Master Pro 451 19-in. CRT monitor (Iyama USA) using Superlab software (Cedrus Inc.).

#### Procedure

**Fig. 5:**
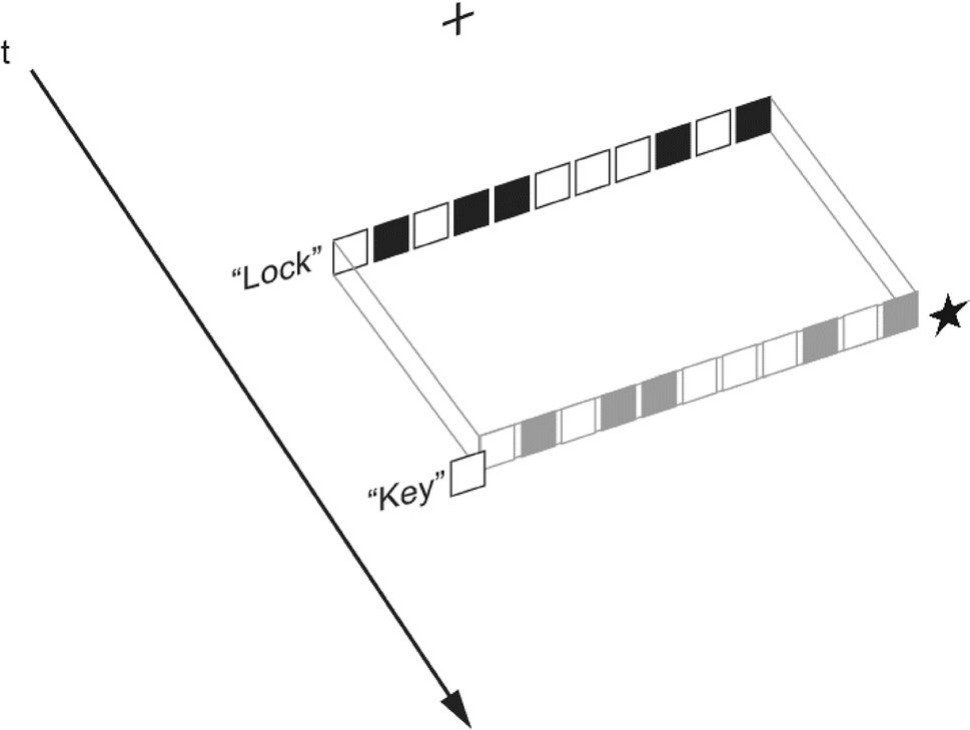
Typical trial structure in Experiment 2. The 11-cell core or “lock” is replaced by a two-element “key” which completes the pattern. In this case, the entire (lock + key) pattern is a CS and the target is a black star located on the right-hand side

Experiments 2, 3 and 4 were run in a dedicated research cubicle and within a single 1-h session. The order of experiments was counterbalanced across subjects using a Latin Square design. After providing consent, participants were shown on-screen instructions and initiated the experiment. Each trial started with a central cross-presented for 750 ms. Following this, an 11-element “lock” was presented for 750 or 1500 ms and then extinguished to be replaced by the (response-extinguished) “key” (Fig. 5). The target itself was a small pentagram, roughly the area of a single pattern square. Its colour (black, white) and location (left, right) were freely varied. The task was to detect the location of the target star by pressing one of the two keys. Each set of 24 distinct trials was doubled and randomised within a block, giving four blocks and 192 trials per participant. The order of trials in a session was randomised beforehand using a random number generator on the site random.org (Haahr, 2020). An individual session lasted approximately 15 min.

### Results and discussion

Despite the decision to remove the lock before the presentation of the key, overall response accuracy was exceptionally high (0.9% error) rendering the analysis of accuracy data superfluous. We predicted a null effect of symmetry on accuracy because of the absence of the systematic lateral bias in the lock but did not envisage such a ceiling effect. However, this was not detrimental because we hypothesised that the tendency towards a lateral response would be inhibited in the case of a CS stimulus simply because the balanced structure of the completed pattern would make it more difficult to trigger a lateral bias predicated on asymmetrical features. We examined performance speed by means of a four-way repeated-measures ANOVA with variables duration (750, 1500 ms), target side (left, right), black side (left, right) and CS (yes, no) and recorded three significant results. The observed power for all significant effects exceeded 0.80. First, target colour had a significant influence on how fast it was located. A disordinate interaction between target side and black side indicates that targets were more easily detected when they were black. This is not surprising given the increased salience of a black star, $F$(1, 24) = 12.18, $p$ = 0.002, ${\upeta }_{p}^{2}$ = 0.34. Critically, this effect was isolated from what follows.

**Fig. 6:**
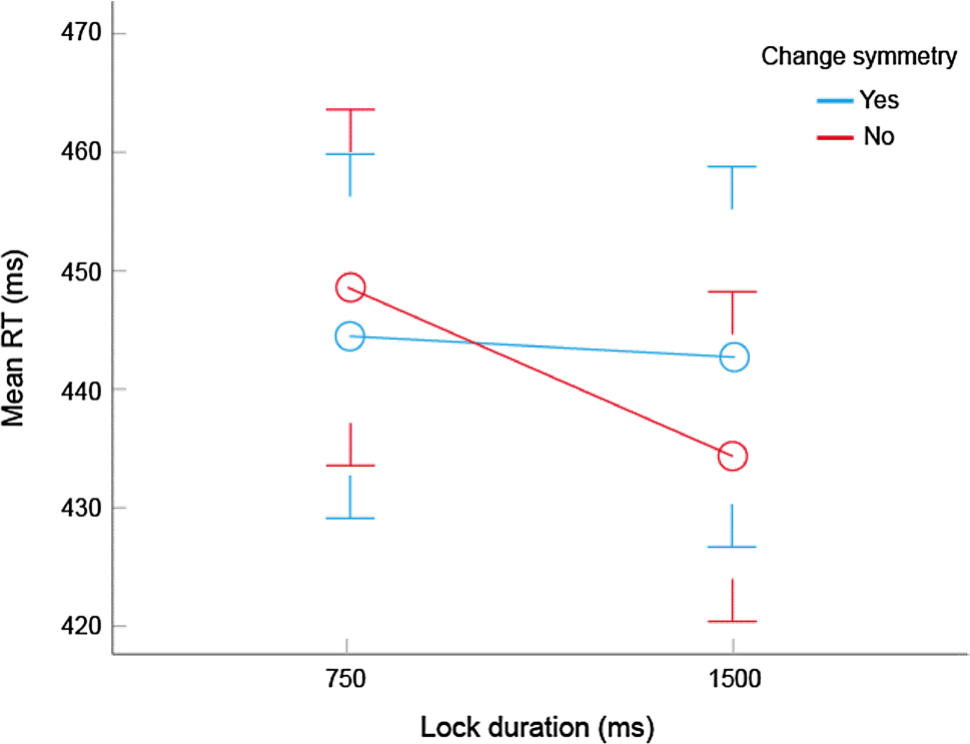
Mean RTs in Experiment 2 as a function of lock duration and CS (± 1 SEM)

There was a significant main effect of duration which suggested that the target registration was facilitated by a longer lock presentation, *F*(1, 24) = 14.83, *p* = 0.001, ${\eta }_{p}^{2}$ = 0.38. This was difficult to explain because (a) taken in isolation, the lock does not contain any useful information and (b) the effect shows no symmetry-related bias. The explanation was provided by a significant duration by CS interaction, $F$(1, 24) = 9.46, $p$ = 0.005, ${\eta }_{p}^{2}$ = 0.28 (Fig. 6). As predicted, non-CS patterns slowed down target detection by about 10 ms. The observed effect reinforced the findings of Experiment 1 that in contrast with other types of (overt) symmetry, CS requires a certain time to process even when it is perceived only implicitly. However, additional experiments were needed to confirm this hypothesis.

## Experiment 3: interference priming of 2D symmetry

Although Experiment 2 produced an interesting finding, it would be difficult to make any substantive claims based on a relatively weak effect. While deliberating whether to keep using the lock and key technique, we decided to apply a technique that combines global structural priming with feature-level masking. Since individual CS patterns differ from each other in terms of the position of individual elements/features, a rapid sequential presentation ensures that the only aspect of the pattern that is reinforced is its global structure—which is not overtly perceivable. In Experiment 1, we established that the structure of CS of length 13 becomes detectable somewhere between 750- and 1500-ms exposure. Consequently, we decided to employ a novel priming technique which helps the cognitive system to extract the implicit symmetry information without the aid of feature cues. Interference priming combines the masking of individual stimulus features with the priming of the underlying structure. Note that this is not possible with overt mirror symmetry where the two halves remain visibly identical. We hypothesized that CSs would implicitly prime 2D arrays of differing degrees of symmetry.

### Methods

#### Participants

See method in Experiment 2.

#### Design, stimuli and apparatus

The design was a pairwise comparison between CS and non-CS conditions. The main dependent variable was mean symmetry rating, but the rating times were also analysed.

The idea behind interference priming is that when a CS pattern is presented briefly, the observer has no way of telling whether it possesses this high-level symmetry (see Fig. 4). Rather, they can access only low-level features and crude indicators of structure, such as runs (Ichikawa, 1985). If the pattern is presented at 500 ms, it is highly unlikely that the symmetry would elicit an overt response (Glanzer & Clark, 1962; as shown in Experiment 1, this was not possible even at 750 ms exposure). So, if a CS pattern is rapidly replaced by another one which has a different layout of 1 s and 0 s, the previous layout is masked/overwritten. This represents an additional insurance against overt registration of CS. This repeated erasure of individual feature layouts happens over a relatively long period (12 s), ensuring that any effects of the prime on target perception are not caused by individual features but by the implicit structure defined by CS which is cumulatively built up over stimulus presentations.

The primes were the 24 black and white patterns already employed in Experiment 2. The targets were 48 5 × 5-cell grids which contained different arrangements of nine black disks. They were selected from the original set of 60 stimuli used by Howe (1980). The stimulus set had been created by selecting six “core” patterns and disrupting them by gradually reducing the amount of symmetry. This produced a sequence of 2D arrays ordered from the most to the least symmetrical. For the purpose of this experiment, the 12 most symmetrical patterns were removed to reduce the possibility of a ceiling effect. Stimulus dimensions were 85 × 85 mm (6.02°).

#### Procedure

**Fig. 7:**
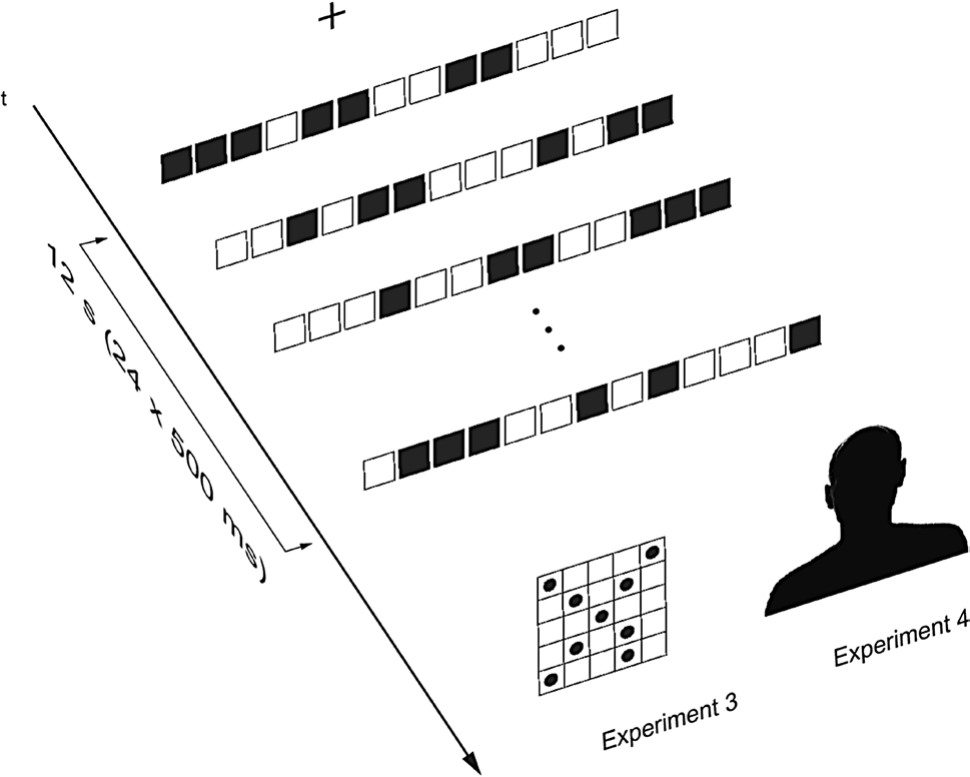
Trial structure in Experiments 3 and 4. A rapid sequence of 24 primes (“interference prime”) is replaced by a target—a 2D grid (Experiment 3) or a face (Experiment 4). The prime stimuli depicted in the figure are CSs

The prime on each trial consisted of a sequence of 24 (2 × 12) bars presented at 500 ms each. The sequence could be either CS or non-CS. The crucial detail here is that the stimulus “burst” does not offer any clues as to the presence of symmetry because each pattern was (a) presented for 500 ms (insufficient for overt recognition) and (b) was quickly replaced by a pattern which contained a different arrangement of black and white squares. This produces two effects—constantly changing pattern features underpinned by an invisible symmetry which persists throughout which ensures that any priming would be due to the (overtly imperceptible) symmetry Gestalt. The order of individual patterns within the sequence was fully randomized. Immediately following the prime (12 s duration), a target was presented and the task consisted of rating the symmetry of the array on a scale from 1 (no symmetry) to 7 (perfect symmetry; see Fig. 7 for trial structure). Responses were made using number keys on the keyboard followed by pressing “Enter” which initiated the next trial. Because of the complexity of the design, individual trials were randomized manually. There were 48 CS and 48 non-CS trials presented within two 48-trial blocks each containing equal numbers of both pattern types. An experimental session lasted approximately 15 min.

### Results and discussion

The difference between CS and non-CS priming conditions was highly significant. The average rating for the former was 4.79 and for the latter, 4.24, *t*(24) = 5.29, *p* < 0.001. The effect size was very large (Cohen’s *d* = 0.095). No difference was observed in terms of rating times. Finally, we compared mean symmetry ratings with AG complexity and obtained a highly significant correlation, *r*(60) = − 0.75, *p* < 0.001. This is close to the correlation with Howe’s ratings (Aksentijevic, Mihailovic, Kapor, et al., 2020). The results reveal two new facts. First, even if convincingly masked, CS exerts measurable influence on perception. Second, 2D symmetries can be successfully primed using 1D stimuli. The primes used in the experiments were 2D, but the symmetry information they carried possessed only one dimension. This suggests the presence of a degree of redundancy in dimensional generalizations of symmetry in that expansion to 2 and 3D is based on a redundant expansion of palindromicity. More importantly, change symmetry holds the promise of becoming a useful tool in the study of symmetry perception. However, such a claim requires additional evidence—in a context that transcends simple feature configurations.

## Experiment 4: priming facial attractiveness

Face perception represents one of the most researched areas in psychology and neuroscience partly because of its importance for basic psychological processes as well as developmental and clinical contexts (Bruce & Young, 2012; Liu, et al., 2010). Facial attractiveness is of particular interest since it has been hypothesized to reflect evolutionary and genetic advantages, and this in turn confers advantages in terms of mate selection, career choice and social advancement (Catena, Simmons & Roney, 2019). Importantly, there is significant agreement that central symmetry can enhance the attractiveness of a face as long as this is not exaggerated (Fink, et al., 2006; Rhodes, et al., 1998). The question that naturally arises is: is it possible to prime positive facial attractiveness ratings by means of symmetric geometrical patterns? In Experiment 4, we went one step further and asked if it was possible to improve facial attractiveness ratings by implicit priming of hidden (change) symmetry.

### Methods

#### Participants

See method in Experiment 2.

#### Design, stimuli and apparatus

The design and experimental parameters were identical to those of Experiment 3. The only important difference concerned target stimuli—in this case, faces. These were extracted from the Chicago face database (CFD; Ma, et al., 2015). This comprehensive repository assesses faces on a large number of subjective and objective variables including perceived attractiveness. This was rated on a 7-point Likert scale (1 = not at all, 7 = extremely). First, we extracted 380 faces with roughly equal representation of gender (male, female) and ethnicity (black, white). Two effects were notable within this sample. Female faces were rated more highly overall, *F*(1, 376) = 17.09, $p$ < 0.001; ${\eta }_{p}^{2}$ = 0.43, and they were rated more highly than black female faces, whereas the opposite was true of male faces, *F*(1, 376) = 4.39, $p$ = 0.37; ${\eta }_{p}^{2}$ = 0.12. Out of this population, we selected 48 faces (12 from each ethnicity/gender combination) such that their attractiveness score was as close to the mean (3.23) as possible. Ethnicity and gender were used to enhance stimulus variability. Our face sample had a mean age of 26.60 years and a mean attractiveness rating of 3.40. The stimuli were colour photographs taken under controlled studio conditions and measured 21.3 × 15.4 cm (15.17 × 11° of visual angle). Trial structure was identical to that of Experiment 3 in that two matched subsets of 24 faces were assigned either to the CS or non-CS condition. The order of presentation of individual faces was randomized manually.

#### Procedure

The procedure was identical to that employed in Experiment 3—the experiment consisted of two 48-trial blocks giving 96 trials in total.

### Results and discussion

**Fig. 8:**
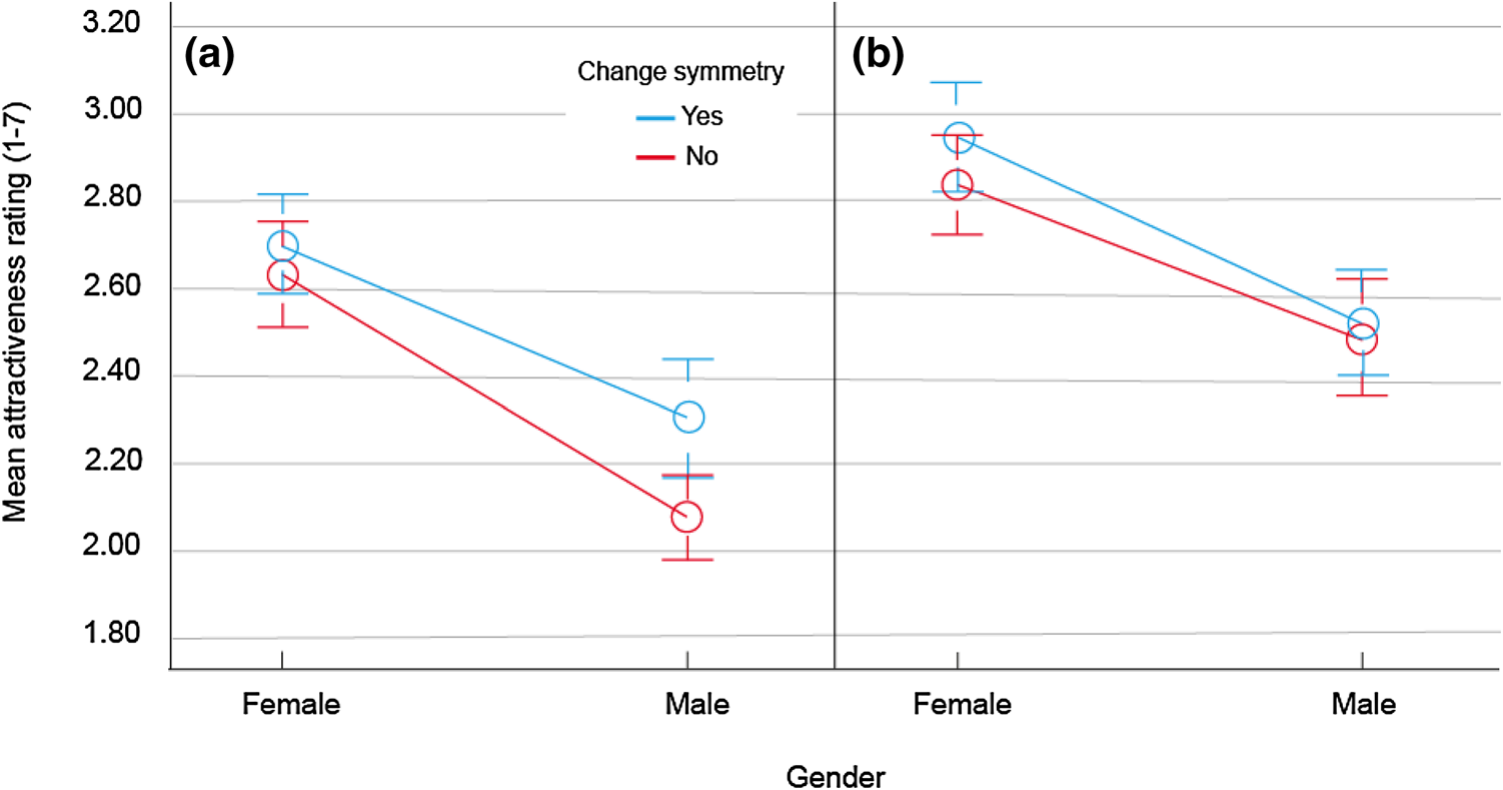
Face attractiveness ratings as a function of ethnicity (**a** = black, **b** = white), gender and the presence of change symmetry (± 1 SEM)

One participant failed to complete the task satisfactorily and their data were removed from analyses. First, we examined the attractiveness rating data. The mean facial attractiveness score across the experiment was lower relative to the normative data (*M* = 2.67). A three-way repeated-measures ANOVA for ethnicity, gender and symmetry revealed significant main effects of these variables, *F*(1, 23) = 23.98, $p$ < 0.001, ${\eta }_{p}^{2}$ = 0.51; *F*(1, 23) = 22.40, $p$ < 0.001, ${\eta }_{p}^{2}$ = 0.49 and *F*(1, 23) = 10.36, $p$ = 0.004, ${\eta }_{p}^{2}$ = 0.31, respectively (Fig. 8). No interactions reached significance. White faces were perceived as more attractive (*M* = 2.69 vs. 2.42) as were female faces (*M* = 2.77 vs. 2.34). Faces primed by CSs were perceived as more attractive relative to those primed with non-CSs (*M* = 2.60 vs. 2.51). The observed power was > 0.80 in all cases. Next, we analysed rating times. These revealed no differences with respect to CS but there was a highly significant (and disordinal) three-way interaction, *F*(1, 23) = 15.80, *p* < 0.001, ${\eta }_{p}^{2}$ < 0.40. Briefly, for black faces, CS facilitated responses to female faces and for white faces, it produced faster responses for male faces.

Although we cannot speculate on the source of this RT effect, both the gender and ethnicity rating effects were predictable from previous research. For example, lower attractiveness ratings for black faces with a predominantly white participant sample are often caused by a lack of familiarity with a statistically rare minority physiognomy. This is supported by a marginally significant RT advantage for black ratings, *F*(1, 23) = 3.85, *p* = 0.062, ${\eta }_{p}^{2}$ = 0.14, suggesting that the participants dwelled slightly longer on the white faces. By contrast, both white and black participants are familiar with the majority white facial features and this often results in higher attractiveness judgments (Coetzee, Greeff, Stephen, & Perrett, 2014). Although this was not observed in the original CFD ratings, the subjects there exhibited a lower discriminability for black male and female faces—another effect of familiarity.

The results of Experiment 4 confirm that facial attractiveness is intimately linked to symmetry and that the latter can impact the perception of the former even when conceptualised in a very abstract way that is (a) completely unrelated to the target stimulus and (b) invisible to the naked eye.

## General discussion

The present study investigated effects of a novel form of symmetry (change symmetry) on the perception of singleton targets, 2D arrays and faces. The concept of change symmetry (or general palindromicity) arises from a shift away from the focus on elements/features/objects to a more holistic Gestalt-like approach which acknowledges the importance of the relationships between objects. While the feature-based approach to perception has resulted in a number of interesting ideas and results, ultimately, it fails to address the most important question, namely how do we perceive the whole? This is clearly much more than a simple/linear agglomeration of features. Although obvious, this insight is not very helpful unless it can be translated into some kind of quantitative theory. Aksentijevic–Gibson complexity is one theory which translates the Gestalt insight into a quantitative model of perception. In the context of the current paper, the most relevant is the conceptual shift from objects to the relationship between them. The most basic relationship is identity/difference. In a dynamic context, this is translatable to change and absence of change, respectively. Aside from its theoretical advantages, using change to describe structure is efficient because it tells us something about the hierarchical ordering of information without having to rely on probability which hampers the quantification of higher structural levels (see e.g. Attneave, 1959).

We predicted that CSs would boost the perceived symmetry either directly or indirectly. To ensure absence of featural symmetry clues, we first established the time needed to perceive CS (Experiment 1) and then applied a couple of novel symmetry priming techniques. In Experiment 2, we applied a lock and key principle to avoid presenting the entire CS prime at the same time. The core of the pattern (lock) is presented and then extinguished to be replaced by the side elements (key). In Experiments 3 and 4, we presented priming stimuli rapidly and sequentially so that even if the symmetrical “essence” of an individual stimulus could be perceived, it would be erased and replaced by the succeeding pattern. In the latter experiments, we employed 2D targets to minimize any figural associations with the prime.

As demonstrated by the pilot experiment, under free-viewing conditions and a long presentation time (3000 ms), difference between CSs and non-CSs could be discerned—non-CS patterns were perceived as somewhat more complex (participants did not spot any structural differences). This compelled us to seek out a point in time at which the two types of pattern became indistinguishable. The results of Experiment 1 suggested that viewers required more than 750 ms to distinguish between them. One way of capitalising on this was to investigate how CS affected performance on an unrelated task (Experiment 2). A CS stimulus was never shown on the screen in its entirety and any effects of symmetry would have been caused by the completion of the pattern in a memory trace. The result confirmed that at 1500-ms duration of the lock segment, the presence of CS slowed down target detection.

To reduce the possibility of confounding by various stimulus-related factors, we designed a prime which was robust with respect to these. First, each priming stimulus was presented for 500 ms—time that is too short to permit the recognition of CS. Next, we created a priming “burst” containing 24 stimuli (two sets of 12 freshly randomised for every trial). This means that every prime is masked and erased by the next one. Added to the brief presentation time, this strategy ensures that information contained within a single priming stimulus cannot affect the participants’ response. All that is left is the “distillate” of high-level structure which is perceived implicitly. This is salient enough to bias the perceptual system even further towards a preference for symmetry.

The result of Experiment 3 can be explained in terms of energetic demands associated with perception and cognition. As elaborated elsewhere (e.g. Treder, 2010), complexity is intimately related to the cost of information processing. In that context, symmetry represents probably the most important redundancy signal in perception. The impact of visual symmetry on the perceiver is partly owed to the fact that a symmetrical form is easy to process, memorise and reproduce (e.g. Reber, Schwarz, & Winkielman, 2004). It could be said that the perceptual system is highly attuned to symmetry by the need to minimise effort (Falk & Konold, 1997). It seeks out symmetries and other informational shortcuts to direct effort towards stimuli that lack such shortcuts. Given the inherent symmetry bias, it is not surprising that presenting symmetric stimuli as primes should strengthen it. What is more interesting is that the presentation of 1D information which is not overtly perceived as symmetrical achieves the same result.

While the results of Experiment 3 are largely understandable if surprising, the findings of Experiment 4 are intriguing and challenging. The importance of facial and bodily symmetry has been linked to evolutionary advantages especially with respect to mate selection (Grammer & Thornhill, 1994; Møller & Thornhill, 1998). These qualities have been linked to a good genetic inheritance and health (Scheib, Gangestad, & Thornhill, 1999). Good health in turn ensures safe procreation and survival of one’s genetic line. Although not very romantic, the evolutionary explanation for the effects of facial symmetry on facial attractiveness is compelling. How then can one explain the fact that such an important evolutionary function is primed by a blurry sequence of black and white squares? Clearly, symmetry, even at a very high, abstract level, guides perception. Although this is not a new observation, the results of the present study demonstrate the profundity and power of its influence. One possible explanation concerns the concept of processing fluency which associates visual symmetry with positive effect that in our case is transferred to faces resulting in higher attractiveness judgment (Pecchinenda, Bertamini, Makin, & Ruta, 2014).

Regarding a specific mechanism, it is too early to speculate although recent work points at a possible way forward. Work by Kietzmann et al. (2017) has shown that the processing of different facial viewpoints produces distinct spatiotemporal patterns of cortical activation. Whereas the first and earliest viewpoint encoding scheme (60 ms post stimulus) encodes separate face orientations, the next one (peaking at approximately 115 ms), mirror symmetrical representations are recognised and the frontal representation is distinguished at about 280 ms post stimulus. FMRI results (e.g. Sasaki, et al., 2005; Tyler et al. 2005) place symmetry perception in the extra-striatal occipital lateral areas (OL). Assuming that CS takes longer to encode than visible symmetry, one could speculate that the “meeting” of symmetry and face-specific activation clusters occurs at approximately 300 ms post stimulus at the transitional area between the occipital lateral and parietal central areas. Once the structural effects of face and symmetry processing are merged, the structurally enhanced representation elicits a higher affective response.

Generally, we can locate our paradigm at the intersection of Gestalt perceptual organisation and hierarchical processing (Han & Humphreys, 1999). The distinction between local and global processing was established by means of suitable stimuli which could be manipulated to accentuate/attenuate either aspect (Navon, 1977). The main difference between Navon’s and our paradigm is the divergence between local asymmetry and global symmetry which enables us to dissociate the two levels more completely. With Navon-type stimuli, both the global and local information is equally available. Second, the function of Navon’s task is to diagnose the type of processing favoured in a particular experimental context. CS, on the other hand, can be used both as a prime and a target. More importantly, CS provides a more realistic definition of global processing in that the global configuration is not a simple sum of local elements (Nucci & Wagemans, 2007).

The observed effects of change symmetry allow us to theorise on the stages of perceptual processing. Very simple patterns (those containing few runs) are easily handled by both local or global strategies. For example, a pattern comprising few elements/clusters can be assimilated rapidly (e.g. black squares on the left; Aksentijevic & Gibson, 2012a; Glanzer & Clark, 1962). This agrees with the finding that grouping by proximity is faster than other forms of grouping (e.g. Ben-Av & Sagi, 1995) and that such Gestalt grouping processes are largely pre-attentional (Baylis & Driver, 1993; Humphreys, Olson, Romani, & Riddoch, 1996; Kahneman & Treisman, 1984; Kramer & Jacobson, 1991; Moore & Egeth, 1997). Here, one should note that a possible confounding with stimulus complexity and also that absence of attention do not necessarily imply conscious perception and categorisation—even when attention is involved. Furthermore, research has demonstrated that attention does not play a significant role in symmetry detection (Huang, et al., 2004) although the situation is not straightforward (Treder, 2010). Whether perfect or partial, symmetry is processed in parallel across the stimulus without capacity limit. This could be explainable by means of a mechanism which assesses incoming stimuli perhaps by means of hard-wired cross-cortical structures which perform comparisons of the two halves of the stimulus (Corballis & Roldan, 1974; Julesz, 1971).

As patterns become more complex, more time is required for their structure to be processed and this is where two-stage theories become relevant. Although it is clear that the brain requires time to absorb the featural arrangement (in agreement with Chipman. 1977; Ichikawa, 1985), we have no evidence that the effects of CS are conscious or based on a cognitive strategy. Rather, we believe that CS accesses the higher areas of the visual brain (V3 and above; Keefe, et al., 2018; Sasaki, et al., 2005; Tyler, Baseler, Kontsevich, Likova, Wade, & Wandell, 2005) extra-attentionally and from there guides the processing of the incoming structural information by looking for changes between the two halves of the stimulus.

## Conclusion

In a series of four experiments, we investigated the priming properties of a novel form of symmetry and found out that it negatively affects lateral target detection (Experiment 2), enhances symmetry ratings for 2D arrays (Experiment 3) and attractiveness ratings for faces of different ethnicities and genders. We observed clear priming effects, especially in Experiments 3 and 4. What are the broader implications of our findings? First, although CS does not have the appearance of symmetry, it behaves like one. It retards lateral target detection (Experiment 2), primes 2D symmetry perception (Experiment 3) and surprisingly, facial attraction (Experiment 4). We suggest that the priming benefits of CS have to do with the parallel processing which captures the *L* – 1 redundancy and treats it as an ordinary mirror symmetry.

One interesting aspect of our results is the dissociation between effects of exposure time and engagement of attention. On the one hand, some effects of CS emerge only after a long exposure (over a second; Experiments 1 and 2). On the other hand, the results of Experiments 3 and 4 suggest an extra-attentional effect by virtue of stimulus design (future research will investigate the precise timing mechanism of CS priming). The results of the first two experiments offer support for a two-stage model of pattern processing, such as Ichikawa’s (1985), according to which the primary stage of processing involves comparisons of surface/quantitative aspects of a pattern. These findings are also in agreement with the proposal by Behrmann and Kimchi (2003) who posit a distinction between low-level visual processes responsible for simple local grouping and higher-level processes responsible for holistic processing. Finally, the results of Experiments 1 and 2 appear compatible with the theorizing by Roelfsema (2006) which distinguishes between two types of grouping mechanism, namely fast “base-grouping” responsible for simple feature extraction and slower incremental grouping which governs higher-level structural processes. As shown by our results, holistic processing of structure and specifically symmetry, affects judgments which can be but do not have to be strictly perceptual. Although we were not able precisely to determine the underlying temporal mechanisms, CS “did its work” only after a period of about 1000–1500 ms. In perceptual research, such durations are commonly associated with conscious perception. However, here, the situation was a bit more complicated in that even prolonged exposure to the prime does not guarantee overt registration of the symmetry.

While Experiments 1 and 2 support two-stage accounts of structure processing, Experiments 3 and 4 indicate that however much time it takes for CS to be encoded, it behaves like a symmetry despite the fact that no symmetry in the accepted sense is present in the stimulus. This poses a question that needs to be addressed by future research, namely is it possible that CS behaves differently from visible symmetry in that its processing is slow yet extra-attentional? Thus, further research is needed to explore the attentional status of change symmetry. More research is also needed to establish a relationship between parameters, such as pattern length and time needed for the symmetry to become overtly available. At the same time, CS could represent a useful tool for probing certain aspects of early perceptual processing especially those pertaining to global vs. local processing. As hypothesized by Aksentijevic and Gibson (2012a), change conceptually precedes invariance and this could translate into “detection of change precedes detection of invariance”, thus allowing precise investigation of the time course of symmetry processing.

In conclusion, we present results of four experiments that demonstrate the effectiveness of a new form of symmetry in producing covert priming effects in three different experimental contexts. This “change symmetry” is not easily perceivable and as such offers a new venue for investigating perceptual processing of structural invariance.

## Figures and Tables

**Fig. 9:**
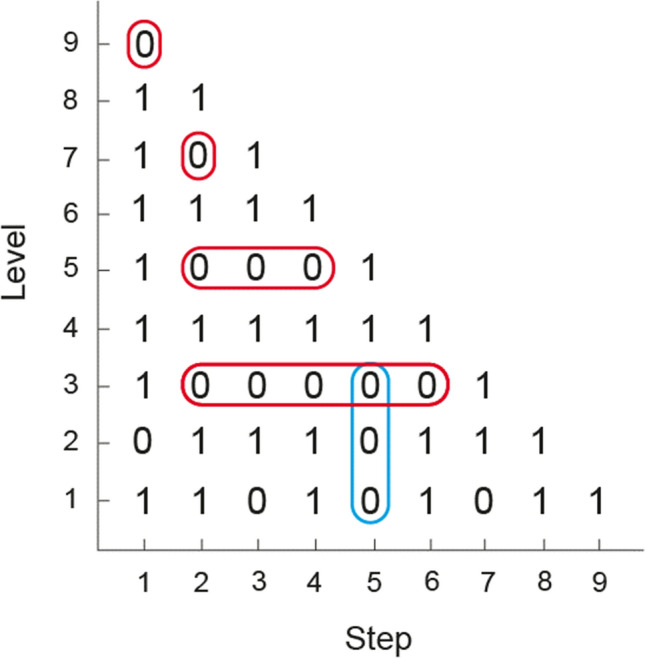
Structure map of the string 1011001101. The abscissa denotes scanning steps and the ordinate successive structural levels. Redundancy within the string leaves two kinds of trace—vertical (blue) and horizontal (red). See text for details (color figure online)

**Fig. 10:**
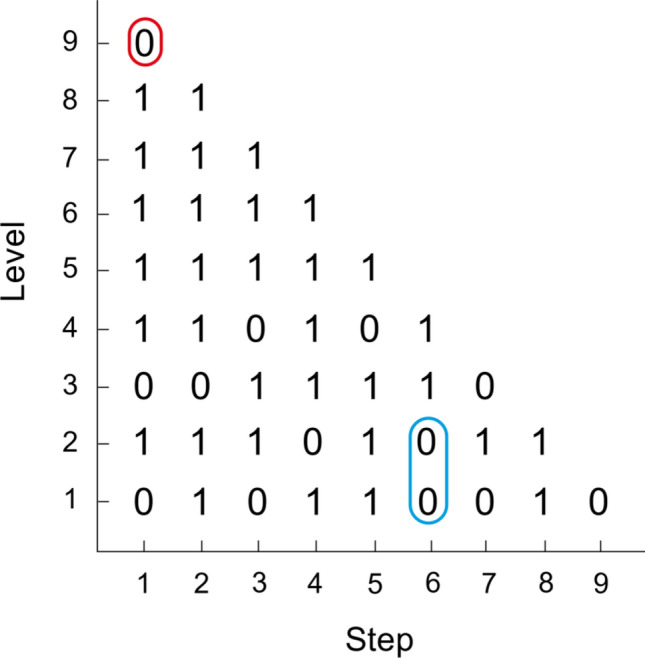
The structure map for the string 0011011100. A small vertical redundancy trace is visible as is the presence of change symmetry (red) (color figure online)

## Appendix 3: Step-by-step guide to detecting change symmetry

As stated in the main body of the text, change symmetries are a consequence of a specific approach to pattern structure based on change. When calculating AG complexity, some strings are visibly symmetrical and they always have a very low complexity. The symmetry can be either rotational or translational (AG requires no more and perhaps less than four periods of any pattern to detect a regularity). However, with change symmetry, the low-level structure is not visibly symmetrical and it is only if one perseveres by comparing different substrings that one might arrive at the rule (which is very difficult to articulate): A change symmetry exists when the two longest overlapping substrings possess identical change profiles. To make this clear, we are going to show here how to detect change symmetry in a string using AG complexity and examine its structure briefly. The easiest way to think of CS is as a hidden or partial or slightly messed up symmetry. It is there, yet it isn’t. For the ease of understanding, we shall start with a visibly symmetrical string: 1011001101.

**Table 4:** Computing a complexity profile for string 1011001101

Level $j$	Differences	Change transform	Number of changes
1	1 ≠ 0 ≠ 1 1 ≠ 0 0 ≠ 1 1 ≠ 0 ≠ 1	110101011	6
2	10 01 ≠ 11 ≠ 10 ≠ 0 0 ≠ 01 ≠ 11 ≠ 10 01	01110111	6
3	101 ≠ 011 110 100 001 011 110 ≠ 101	1000001	2
4	1011 ≠ 0110 ≠ 1100 ≠ 1001 ≠ 0011 ≠ 0110 ≠ 1101	111111	6
5	10110 ≠ 01100 11001 10011 00110 ≠ 01101	10001	2
6	011001 ≠ 011001 ≠ 110011 ≠ 100110 ≠ 001101	1111	4
7	1011001 ≠ 0110011 1100110 ≠ 1001101	101	2
8	101100110 ≠ 01100110 ≠ 1011001101	11	2
9	101100110 011001101	0	0

Only inequalities are labelled for clarity

On the left-hand side of Table 4, you can see the comparisons of substrings of all lengths. Overall, there are $N\times (N-1)/2$ or in this case, 45 scans. Unlike Shannon entropy which relies on the comparison between a sample and a population (which leads to a loss of information due to a combinatorial explosion), AG complexity is “self-limiting” in that the drop in the number of scans with increase in substring length *j* is compensated for by the redundant scanning over all the levels.

The main product of scanning is a “change transform” (third column in Table 4) which documents the location in space and time of every change at every level and translates the original scans into a structure map (see Fig. 9). The steps on the abscissa are equivalent to the steps in a time series with the caveat that higher levels produce fewer scans (time). The levels on the ordinate range from 1 (comparison between symbols) to $L-1$ (the longest substrings; space). The structure map represents the structural “DNA fingerprint” of a string and contains all available information about it to the extent that even a single shifting of a symbol in a string of any length will produce a distinctive change in the map. This means that every structurally distinct string possesses its own unique structure map.^7^

As can be seen in Fig. 9, the string 1011001101 produces clear evidence of redundancy both vertical (between-level; blue) and horizontal (within-level; red). The former shows the spread of a structural regularity through different levels. Those regularities that propagate through several levels often signal the presence of important structures in the data. The within-level redundancy is more common because there is always a possibility of local order within disorder and its importance depends on the relative length of the no-change run and possible between-level corroboration.^8^ In our example, long runs of zeros within levels repeat every two levels indicating that there is more to this than just chance (we know that it is because unlike a scientist examining data, we are aware of the global symmetry of the string).

However powerful this informational snapshot is, it creates a challenge. A structure map is too rich to provide a single scalar complexity value. As shown elsewhere, it does furnish a very powerful and detailed image of the structure of a string and perhaps that is enough. Fortunately, it is possible to convert the map into a single-value complexity measure via two simple steps. First, we have to ignore the temporal location of changes and consider their quantity only (see rightmost column in Table 4). When the tallies from individual levels are ordered from left to right (lowest to highest level), we obtain a complexity profile of the string: 6 6 2 6 2 4 2 2 0. The loss of positional information makes this representation more compact and malleable. The final step of this procedure is explained in the introduction (Fig. 10).

To demonstrate the detection of a change symmetry we shall use the string 0011011100 ($L$ = 10). What is immediately obvious in Fig. 10 is the absence of large regions of no-change. However, the appearance is deceptive. Highlighted in red is the reason for the current paper, namely the presence of change symmetry which is signalled by the absence of change between the two longest (overlapping) substrings “00110110” and “011011100”.^9^ Shifting the scanning head from the former to the latter cannot detect any changes because their change profiles are identical (45434321) as are their complexities (6.48). In other words, AG is blind to what it sees as a non-structural difference between the two substrings.

Thank you and now feel free to return to the introduction!

## Footnotes

1 Perceived or subjective complexity refers to human complexity judgments elicited in psychological experiments.

2 To understand how change profiles are derived and change symmetry detected, please consult Appendix 3.

3 While some authors refer to “imperfect symmetry” (e.g. Huang et al., 2004), we avoid using this term because we consider CS to be perfect in terms of change (see Fig. 1).

4 31 participants (15 male) took part. 20 patterns (10 CSs and 10 non-CSs) were presented randomly via a Microsoft Power Point slideshow. Patterns, visually identical to those used in Experiment 1, were presented in the centre of the display for 3000 ms. Participants verbally judged the complexity of a pattern on a 6-point scale from “very simple” to “very complex”. Non-CS ratings were significantly higher relative to CS ratings, *t*(30) = 2.38, *p* = 0.024.

5 From the perspective of AG complexity, the 232 perceptually distinct strings are divided into 58 classes of structurally distinct strings with four members each. Each class consists of an original string, its mirror inversion, complement and mirror complement.
